# Supplementary material for: In Vitro and In Vivo Human Body Odor Analysis Method Using GO:PANI/ZNRs/ZIF−8 Adsorbent Followed by GC/MS
Source: Molecules. 2022 Jul 27;27(15):4795. doi: 10.3390/molecules27154795 (PMC9369976; doi:10.3390/molecules27154795)
Supplement: Supplementary file 1 [file molecules-27-04795-s001.zip › molecules-1729764-supplementary.pdf]

Supplementary materials for

In-Vitro and In-Vivo Human Body Odor Analysis Method using GO;PANI/ZNRs/ZIF−8 adsorbent followed by GC/MS

Sehyun Kim, Sunyoung Bae*

Department of Chemistry, Seoul Women's University, 621 Hwarang-ro, Nowon-gu, Seoul 01797, Republic of Korea

*Corresponding Author

Email: sbae@swu.ac.kr

S1. Experimental

Chemicals and Instrumentation

Sulfuric acid (Samchun, Seoul, Korea, 95.0%), sodium nitrate (Sigma-Aldrich, St. Louis, Missouri, USA, >99.0%), potassium permanganate (Samchun, Seoul, Korea, 99.3%), hydrogen peroxide (Daejung, Gyeonggi-do, Korea, 30%), hydrochloric acid (Samchun, Seoul, Korea, 35.0 ~ 37.0%) were used to oxidize natural graphite powder (30 μm, 99.9%) from US Research Nanomaterials, Inc (Houston, USA). Aniline (>99.5%), zinc nitrate hexahydrate (98%), hexamethylenetetramine (99%), 2-methyl imidazole (99%), and N,N-dimethylformamide (>99.8%) were purchased from Sigma-Aldrich (St. Louis, Missouri, USA) to make GO;PANI/ZNRs/ZIF−8 coating layer on stainless steel wire

O

Table S1. Physicochemical properties of target compounds

Compound	Structure	Molecular weight (g/mol)	Boiling point (°C)	$logP_{o/w}$
trans-2-Nonenal	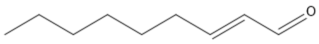	140.23	188~190	3.06
Benzothiazole	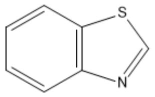	135.19	227~228	2.01
Isopropyl palmitate	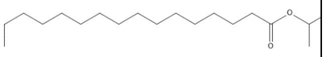	298.5	160	7.17
α-Hexyl cinnamaldehyde	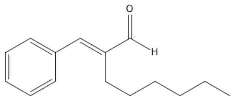	216.32	174~176	4.70
Hexyl salicylate	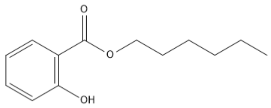	222.28	290	5.06

O

Optimization using response surface methodology (RSM)

Table S2. Experimental design matrix for optimization of reactants concentration in fabrication of adsorbent

Run	Coded factor		
	GO	Aniline	2-MI
1	-1	0	1
2	0	0	1
3	1	0	1
4	-1	-1	1
5	-1	1	1
6	-1	1	-1
7	-1	0	0
8	-1	1	-1
9	-1	1	-1
10	-1	1	-1
11	-1	1	-1
12	1	0	1
13	0	0	0
14	0	1	1
15	-1	-1	-1
16	1	1	-1
17	-1	-1	0

O

Table S3. Experimental design matrix for optimization of HS-INME condition

Run	Coded factor		
	Extraction temp.	Adsorption time	Desorption time
1	-1	0	1
2	0	0	1
3	1	0	1
4	-1	-1	1
5	-1	1	1
6	-1	1	-1
7	-1	0	0
8	-1	1	-1
9	-1	1	-1
10	-1	1	-1
11	-1	1	-1
12	1	0	1
13	0	0	0
14	0	1	1
15	-1	-1	-1
16	1	1	-1
17	-1	-1	0

O

S2. Optimization of body odor adsorbent fabrication conditions using RSM

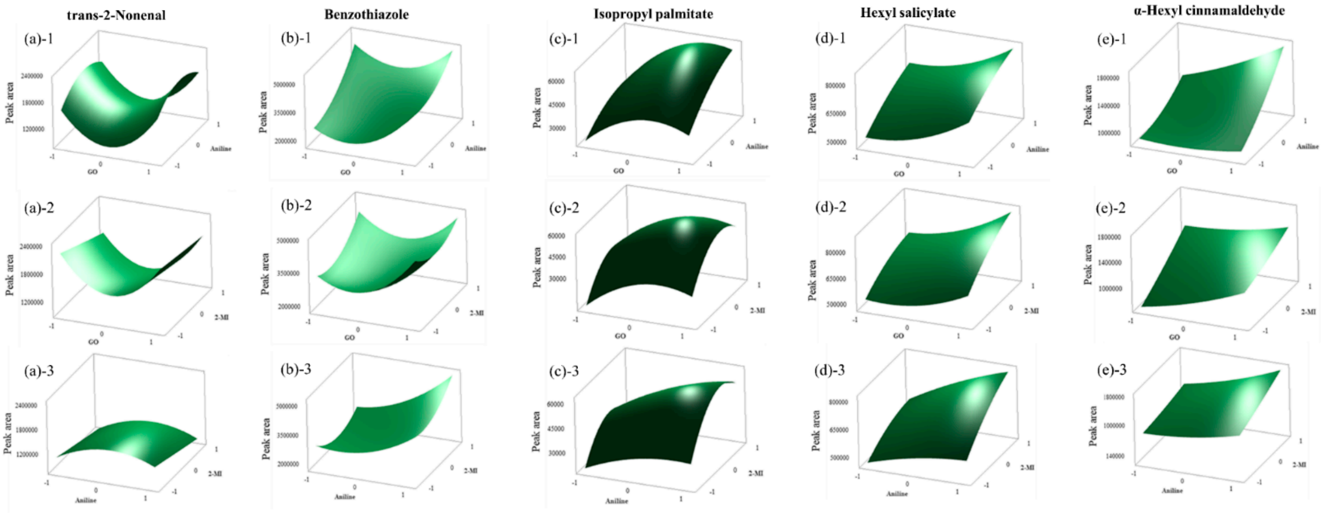

Figure S1. Response surface diagrams of target compounds by factors; (a) *trans*-2-nonenal, (b) benzothiazole, (c) isopropyl palmitate, (d) hexyl salicylate, (e) α-hexyl cinnamaldehyde, and correlation between 1: amount of GO and amount of aniline, 2: amount of GO and amount of 2-MI, and 3: amount of aniline and amount of 2-MI.

S3. Optimization of INME extraction conditions using RSM

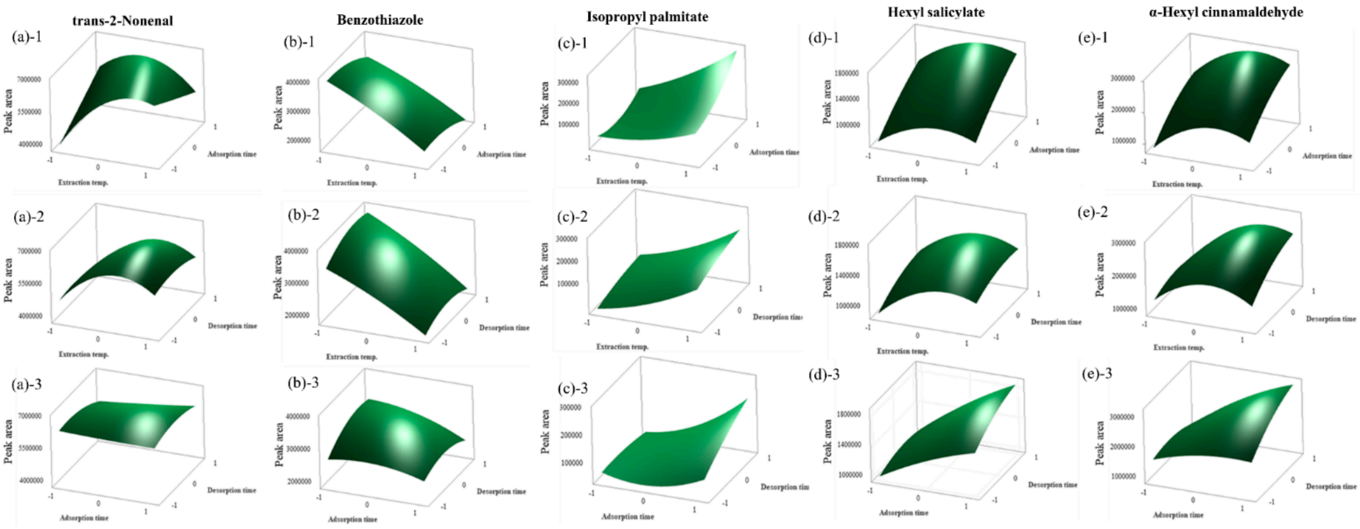

Figure S2. Response surface diagrams of target compounds by factors; (a) *trans*-2-nonenal, (b) benzothiazole, (c) isopropyl palmitate, (d) hexyl salicylate, (e) α-hexyl cinnamaldehyde and correlation between 1: extraction temp. and adsorption time, 2: extraction temp. and desorption time, and 3: adsorption time and desorption time.

S4. Characterization of the adsorbent

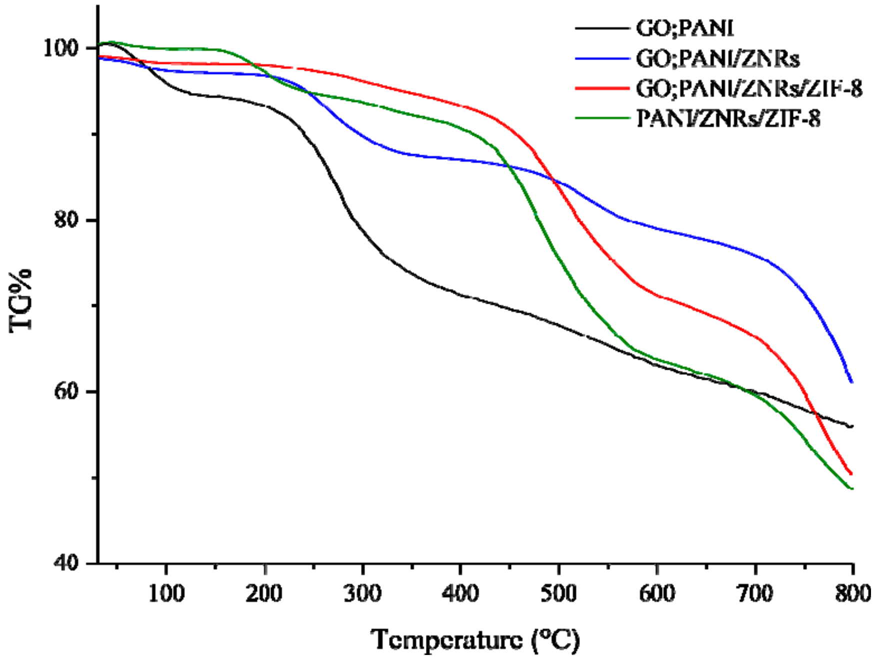

Figure S3. TGA curves of GO;PANI, GO;PANI/ZNRs, GO;PANI/ZNRs/ZIF−8, and PANI/ZNRs/ZIF−8.

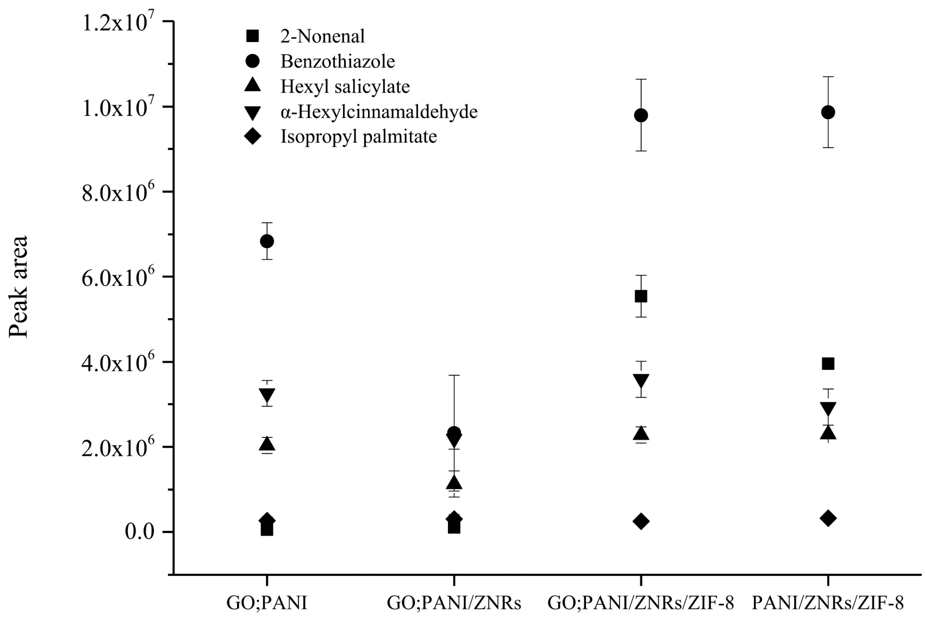

Figure S4. Comparison of adsorption amount of five target compounds according to the adsorbent synthesis steps, and without GO.
